# Solutions of a Linearized Mathematical Model for Capillary Formation in Tumor Angiogenesis: An Initial Data Perturbation Approximation

**DOI:** 10.1155/2013/789402

**Published:** 2013-08-18

**Authors:** Serdal Pamuk

**Affiliations:** Department of Mathematics, University of Kocaeli, Umuttepe Campus, Kocaeli, 41380 Izmit, Turkey

## Abstract

We present a mathematical model for capillary formation in tumor angiogenesis and solve it by linearizing it using an initial data perturbation method. This method is highly effective to obtain solutions of nonlinear coupled differential equations. We also provide a specific example resulting, that even a few terms of the obtained series solutions are enough to have an idea for the endothelial cell movement in a capillary. MATLAB-generated figures are provided, and the stability criteria are determined for the steady-state solution of the cell equation.

## 1. Introduction

Angiogenesis is the main feature of neovascularization, the formation of new blood vessels. It is defined as the outgrowth of new vessels from a preexisting vascular network and is fundamental to the formation of blood vessels during placental growth and wound healing, for example. It is also known that it occurs in three sequential steps [1]. First, the endothelial cells (EC) lining the vascular basal lamina (BL) (or basement membrane) degrade this membrane. Second, the EC migrate and proliferate (via mitosis) into the extracellular matrix (ECM). Finally, capillary loops form. In recent years, progress has been made to understand this phenomenon at the molecular level. This includes the identification of potent angiogenic factors, the discovery of the role of proteases, the importance of the ECM, and the emerging characterization of signal transduction pathways in EC.

One of the major components of the ECM is fibronectin, a large, highly adhesive glycoprotein particularly abundant in plasma, connective tissue matrices, and BL [2]. It is also known to enhance EC adhesion to collagen and is produced by EC [3]. The simplest unifying interpretation of these findings is that it functions as adhesive protein that binds cells to other cells or to substrate. Fibronectin-treated cells also migrate more rapidly, both as single cells or as masses of cells migrating out from cell aggregates [3].

As stated in [4, 5] EC are to be stimulated by a tumor angiogenic factor for angiogenesis to occur. Also, active enzyme stimulates the EC migration [6]. Once the EC are stimulated, the long-time tendency of them is towards the transition probability density function (TPDF) [7] of active enzyme and fibronectin (see [8] for mathematical proof of this).

Endothelial cell migration and proliferation also occur during endothelial repair *in situ*; the ability to penetrate the vascular basement membrane, on the other hand, is an aspect of endothelial cell behavior uniquely expressed during angiogenesis [9].

There have been many mathematical models describing tumor angiogenesis (see [10–13] and references therein). For example, in [11] the authors propose a review and critical analysis of the asymptotic limit methods focused on the derivation of macroscopic equations for a class of equations modeling complex multicellular systems by methods of the kinetic theory for active particles, and in [12] the authors deal with the derivation of macroscopic equations of biological tissues for a class of nonlinear equations, with quadratic type nonlinearity, modeling complex multicellular systems. Also, in [13] a continuous model for three early stage events in angiogenesis, initiation, sprout extension, and vessel maturation, is presented, and in [8, 14], a mathematical model for capillary network formation is presented, and a mathematical analysis of it is given in one and two dimensions, respectively. The mathematical analyses there were very useful to understand mathematically the mechanism of the cell movement in a tissue (see the biological overview in there). On the other hand, in [15] the authors study some qualitative properties of the solutions of a nonlinear flux-limited equation arising in the transport of morphogens in biological systems, and in [16], to eliminate nonbiological behaviors from diffusion models the authors introduce flux-limited spreading, which implies a restricted velocity for morphogen propagation and a nonlinear mechanism of transport.

The layout of the rest of the paper is as follows. First, we describe our modeling assumptions, and write down our one-dimensional model equations originally presented in [17] in detail. Second, we linearize our model using an initial data perturbation method and solve it. Third, we provide a specific example and plot the results. Finally, we close the paper by presenting the conclusions and discussions on our analysis and the biological importance of our results.

## 2. The Mechanism for the Production of Protease

 If *V* denotes a molecule of angiogenic factor (substrate) and *R*_*E*_ denotes a receptor on the endothelial cell wall, they combine to produce an intermediate complex, *R*_*E*_ *V* which is an activated state of the receptor that results in the production and secretion of proteolytic enzyme, *C*, and a modified intermediate receptor *R*_*E*_′. The receptor *R*_*E*_′ is subsequently removed from the surface by a mechanism that is presumed to be very fast in the time scale of the production of protease *C*. The receptor *R*_*E*_′ is then either recycled back to the cell surface to again become *R*_*E*_ or degraded and new *R*_*E*_ is synthesized, which then moves to the cell surface to replace the *R*_*E*_ that had been removed [8].

The point of view is that the receptors at the surface of the cell function in the same way an enzyme functions in classical enzymatic catalysis [8]. In symbols,

(1) V+RE⇌[REV][REV]→C+RE.

## 3. The Mechanism for the Degradation of Fibronectin

 The decay of fibronectin (*F*) via protease is assumed to satisfy a reaction mechanism of the form [8],

(2) C+F⇌[CF][CF]→C+F′

which shows that *C* acts as a catalyst to convert the fibronectin into products *F*′.

## 4. Modeling Assumptions

**Figure 1:**
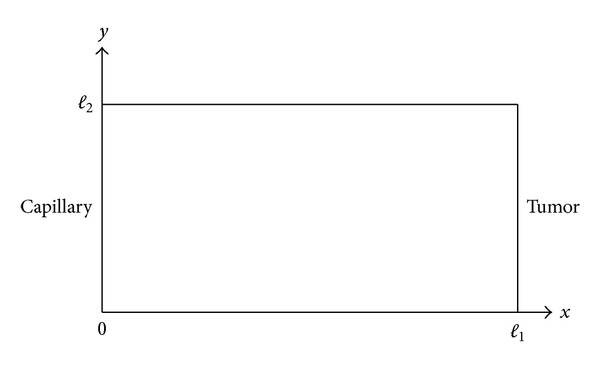
Extracellular matrix (ECM).

 In the *x* − *y* plane we envisage a capillary segment of length *ℓ*_2_ microns located along the *y*-axis on the interval [0, *ℓ*_2_] with a tumor source located somewhere along the line *x* = *ℓ*_1_ (Figure 1) [8]. We rescale *x* by *x*/*ℓ*_1_ and *y* by *y*/*ℓ*_2_ so that this rectangle becomes a unit square. Therefore, we now have 0 ≤ *x*, *y* ≤ 1. Basically, the problem consists of two parts: (i) the dynamics on the *y* axis, namely, in the capillary (1D problem); (ii) the dynamics in the unit square, namely, in the ECM (2D problem). We couple those two dynamics via some boundary conditions (see [17] for details). The 1D problem is the focus of this paper.

## 5. The Model

 We use the following notation for the concentrations of the various chemical species along the capillary wall in *μ*M (micromoles per cubic liter):

(3) v(y,t)=angiogenic  factor  density,c(y,t)=proteolytic  enzyme  density,f(y,t)=fibronectin  density,η(y,t)=endothelial  cell  density.

If we apply the law of mass action to (1)-(2) (see [17] for details), which asserts that the rate of reaction is proportional to the product of the concentrations of the reactants, we obtain the following system of equations:

(4) ∂v∂t=−λv1+νvηη0,∂c∂t=λv1+νvηη0,∂f∂t=βf(1−ff0)ηη0−μcf,

where *λ*, *ν* are kinetic parameters, *η*_0_, *f*_0_ are some reference numbers for EC and fibronectin, respectively, and *β*, *μ* are some positive constants.

Furthermore, if we use the theory of reinforced random walk derived by Davis [18], we obtain the EC movement equation (see [17] for the derivation of the following equation) as follows:

(5) ∂η∂t=Dη∂∂y(η∂∂y(ln⁡ητ^)),

where *D*_*η*_ is a positive constant, the EC diffusion coefficient in the capillary, and $\hat{\tau }$ is the so-called transition probability function. This function has the effect of biasing the random walk of endothelial cells. In this case, we know that the walk is influenced by the proteolytic enzyme it produces in response to the angiogenic factor that has made its way to the cell receptors, and by the fibronectin in the BL, thus, we write

(6) τ^=τ^(c,f).

A simple transition probability which reflects the influence of enzyme and fibronectin on the motion of endothelial cells is $\hat{\tau }(c,f)=c^{\gamma_{1}}f^{-\gamma_{2}}$ for positive constants *γ*_*i*_  (*i* = 1,2).

The biological interpretation of this choice is that endothelial cells prefer to move into the regions where *c* is large or where *f* is small. 

 In order to avoid singularities in $\ln\hat{\tau }$ and its derivatives in (5), it is useful to take

(7) τ^(c,f)=τ1(c)τ2(f),

where

(8) τ1(c)=(α1+cα2+c)γ1,  τ2(f)=(β1+fβ2+f)γ2.

Here the *α*_*i*_, *β*_*i*_, *γ*_*i*_  (*i* = 1,2) are positive constants such that 0 < *α*_1_ ≪ 1 < *α*_2_ and *β*_1_ > 1 ≫ *β*_2_ > 0. Clearly, $\hat{\tau }$ is not singular for small or large values of *c*, *f* and will approximate *c* ^*γ*_1_^ *f* ^−*γ*_2_^ over a considerable range of these variables [17].

We impose zero flux boundary conditions for the cells in (5)

(9) Dηη∂∂yln⁡(ητ^(c,f))=0 (at  y=0,1)

and take

(10) η(y,0)=η0>0,

since we assume that the capillary is initially in a rest state.

## 6. Solutions of the Linearized Model

We first note that we can write (5) as

(11) ∂η∂t=Dη∂∂y(ηy−ητ1′τ1cy−ητ2′τ2fy),

where

(12) τ1′τ1=γ1α2−α1(α1+c)(α2+c),τ2′τ2=γ2β2−β1(β1+f)(β2+f).

We look for the linearized solutions of our model using a perturbation of the initial data in the form *η*(*y*, 0) = *η*_0_ = 1, *v*(*y*, 0) = *ϵθ*(*y*), *c*(*y*, 0) = *c*_0_ = 0, *f*(*y*, 0) = *f*_0_ = 1. Here *ϵ* is some positive parameter and *ϵθ*(*y*) is some initial disturbance in growth factor concentration normalized in such a way that [19, 20]

(13) ∫01θ(y)dy=1.

Correspondingly let us write:

(14) v(y,t)=ϵw(y,t,ϵ),c(y,t)=ϵξ(y,t,ϵ),f(y,t)=1−ϵφ(y,t,ϵ),η(y,t)=1+ϵn(y,t,ϵ).

If we now plug (14) into (4) and (11), respectively, we obtain the following equations

(15) ϵwt=−λϵw(1+ϵn)1+νϵw,ϵξt=λϵw(1+ϵn)1+νϵw,−ϵφt=βϵφ(1−ϵφ)(1+ϵn)−μ(1−ϵφ)ϵξ,ϵnt=Dη∂∂y[ϵny−(1+ϵn)γ1(α2−α1)ϵξy(α1+ϵξ)(α2+ϵξ)    −(1+ϵn)γ2(β2−β1)ϵφy(β1+1−ϵφ)(β2+1−ϵφ)].

If one lets *ɛ* → 0 in (15), our linearized model becomes

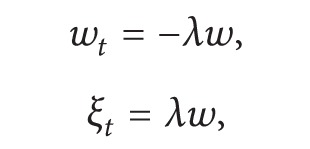
(16)

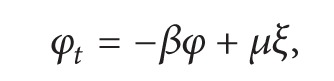
(17)

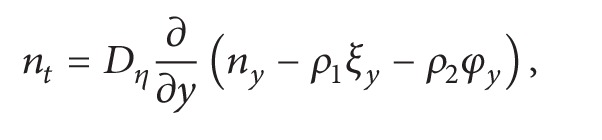
(18)
where

(19) ρ1=γ1(α2−α1)α1α2,  ρ2=γ2(β2−β1)(β1+1)(β2+1).

One easily obtains from (16) that

(20) w(y,t,ϵ)=θ(y)exp⁡(−λt),ξ(y,t,ϵ)=θ(y)(1−exp⁡(−λt)).

If we plug this *ξ* in (17), we obtain

(21) φt+βφ=μθ(y)(1−exp⁡(−λt)),

which is a linear equation in the variable *φ*. The solution to this equation with the initial condition *φ*(*y*, 0, *ϵ*) = 0 is

(22) φ(y,t,ϵ)=μθ(y)[1β−exp⁡⁡(−λt)β−λ+λexp⁡⁡(−βt)β(β−λ)].

If we now write these variables *ξ* and *φ* in (18), it reads

(23) nt=Dη[nyy−(ρ1(1−exp⁡(−λt))       +ρ2μ(1β−exp⁡⁡(−λt)β−λ+λexp⁡⁡(−βt)β(β−λ)))   × θ′′(y)].

For sufficiently large *t* one obtains

(24) nt=Dη[nyy−Kθ′′(y)],

where *K* = *ρ*_1_ + *ρ*_2_ *μ*/*β*.

Using the new variables our boundary conditions in (9) become

(25) ny=ρ1ξy+ρ2φy (at  y=0,1).

Since we want the function *θ*(*y*) to be of unimodal distribution type, it is reasonable to choose *θ*′(0) = *θ*′(1) = 0. Therefore, the conditions in (25) now read

(26) ny=0 (at  y=0,1),

and we come up with the following initial-boundary value problem:

(27) nt−Dηnyy=−DηKθ′′(y),n(y,0,ϵ)=0, 0<y<1,ny(0,t,ϵ)=ny(1,t,ϵ)=0, t>0.

We now let *M*(*y*, *t*, *ϵ*) be any known function and [21]

(28) v(y,t,ϵ)=n(y,t,ϵ)−M(y,t,ϵ).

If we plug (28) in (27), we obtain

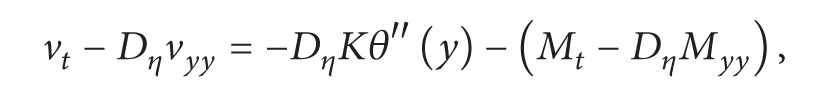
(29)

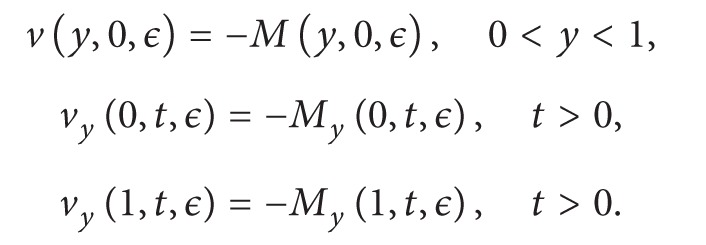
(30)
We now choose *M*(*y*, *t*, *ϵ*) = *Kθ*(*y*) to make (29) homogenous. Therefore, since *θ*′(0) = *θ*′(1) = 0, (29)-(30) become

(31) vt−Dηvyy=0,

(32) $$\begin{array}{c} v\left(y,0,\epsilon \right)=-K\theta \left(y\right),\;0<y<1, \end{array}$$

(33) $$\begin{array}{c} v_{y}\left(0,t,\epsilon \right)=v_{y}\left(1,t,\epsilon \right)=0,\;t>0. \end{array}$$

It is known that this problem has a series solution of the form

(34) v(y,t,ϵ)=C02+∑n=1∞Cne−n2π2Dηtcos⁡(nπy),    0≤y≤1,  t>0.

Letting *t* = 0 in (34) and using (32) give

(35) v(y,0,ϵ)=−Kθ(y)=C02+∑n=1∞Cncos⁡(nπy),   0≤y≤1,

where

(36) C0=−2K∫01θ(y)dy=−2K,Cn=−2K∫01θ(y)cos⁡(nπy)dy, n=1,2,….

Therefore, the solution to the problem given by (27) becomes

(37) n(y,t,ϵ)=C02+∑n=1∞Cne−n2π2Dηtcos⁡(nπy)+Kθ(y),   0≤y≤1,  t>0,

where *C*_0_ and *C*_*n*_'s are the same as those mentioned before.

Consequently, from (14) the perturbation solutions of the system consisting of (4)-(5) together with the initial and boundary conditions are obtained as follows:

(38) v(y,t)=ϵθ(y)exp⁡(−λt),   0<y<1,  t>0,c(y,t)=ϵθ(y)(1−exp⁡(−λt)), 0<y<1,  t>0,f(y,t)=1−ϵμθ(y)[1β−exp⁡⁡(−λt)β−λ+λexp⁡⁡(−βt)β(β−λ)],         0<y<1,  t>0,η(y,t)=1+ϵC02+ϵ∑n=1∞Cne−n2π2Dηtcos⁡(nπy)+ϵKθ(y), 0≤y≤1,  t>0.

Again, all of the constants and parameters seen in (38) are the same as those mentioned before.

## 7. Numerical Example

For numerical purposes we take *θ*(*y*) = *Ay* ^2^(1 − *y*)^2^. By the aid of (13) one has *A* = 30. Also we find *C*_0_ = −2*K* and

(39) Cn={0when  n=1,3,5,…,1440Kn4π4when  n=2,4,6….

Therefore, we have

(40) v(y,t,ϵ)=−K[1−90π4∑n=1∞cos⁡⁡(2nπy)n4e−4n2π2Dηt],            0<y<1,  t>0,

(41) n(y,t,ϵ)=−K[1−90π4∑n=1∞cos⁡⁡(2nπy)n4e−4n2π2Dηt]+30Ky2(1−y)2, 0<y<1,   t>0.

On the other hand, from (20) and (22) one has

(42) w(y,t,ϵ)=30y2(1−y)2exp⁡(−λt), 0<y<1,  t>0,ξ(y,t,ϵ)=30y2(1−y)2(1−exp⁡(−λt)),       0<y<1,   t>0,φ(y,t,ϵ)=30μy2(1−y)2×[1β−exp⁡⁡(−λt)β−λ+λexp⁡⁡(−βt)β(β−λ)],           0<y<1,  t>0.

As a result, the perturbation solutions of the system consisting of (4)-(5) for this special choice of *θ*(*y*) are obtained by plugging (41)-(42) into (14), and they are

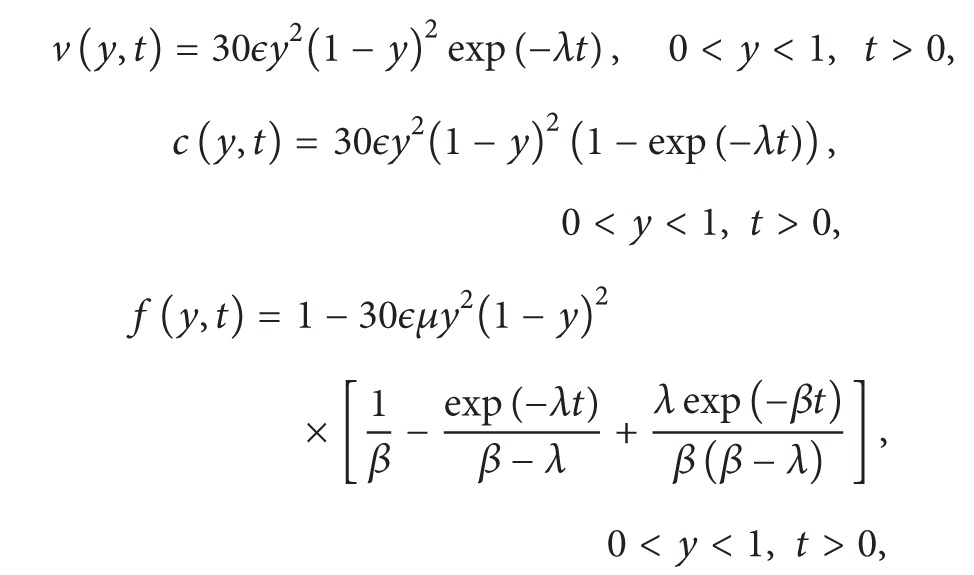
(43)

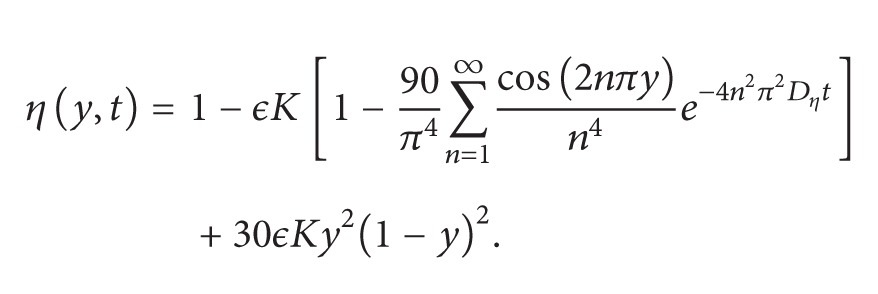
(44)
In this example, we take *ϵ* = 0.05, *λ* = 20, *μ* = 5, *β* = 10, *K* = 0.1, and *D*_*η*_ = 0.25 for our computations. In the following the reader finds the pictures of angiogenic factor, proteolytic enzyme, fibronectin, and endothelial cell densities, respectively, obtained from (43)-(44). The pictures are plotted until *t* = 0.1.

On the other hand, one easily gets from (41) that

(45) lim⁡t→∞n(y,t,ϵ)=−K+30Ky2(1−y)2=−K+Kθ(y).

Also, if we set *n*_*t*_ = 0 in (24), we obtain the steady-state solution as

(46) n(y,ϵ)=B+Kθ(y),

where *B* is an arbitrary constant. Comparison of (45) and (46) gives that this steady state is stable if *B* is taken to be the constant −*K*, which is −0.1 in this case.

## 8. Conclusion and Discussion

In this paper we have presented a mathematical model for capillary formation in tumor angiogenesis and solved it by linearizing it using an initial data perturbation method. Even though our results here are almost the same as those obtained in [17, 19], where this model has been solved by a classical explicit method and by the method of lines, respectively, we believe the linearization method is much more easier and effective.

This model is based on the cell biologist's observations concerning cell movement. In the case of endothelial cells, these are more likely to move places where fibronectin density is low to follow a chemical trail consisting of growth factor and to respond to growth factor by moving into new space created by an enzyme they produce that in turn destroys fibronectin as well as other ECM components [19].

Figures 3, 4, and 5 show the angiogenic factor, proteolytic enzyme, and fibronectin densities, respectively. We have used only four-term expansion of the series in (44) to draw Figure 6. Even with four-term expansion we have obtained what we expect to see for the endothelial cell movement in a capillary. This also shows the effectiveness of our method. Of course, a more stable solution for the cell equation can be obtained by expanding more terms of the series solution.

**Figure 2:**
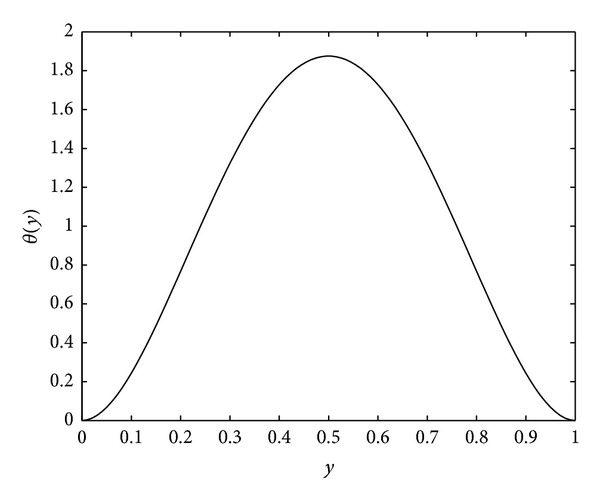
Profile of possible unimodal distribution of *θ*(*y*).

**Figure 3:**
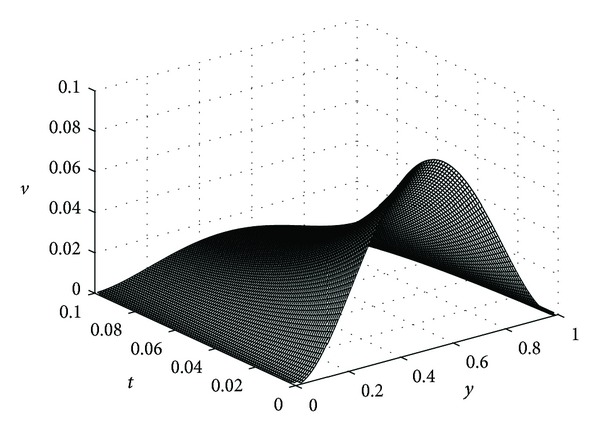
Angiogenic factor density *v*(*y*, *t*).

**Figure 4:**
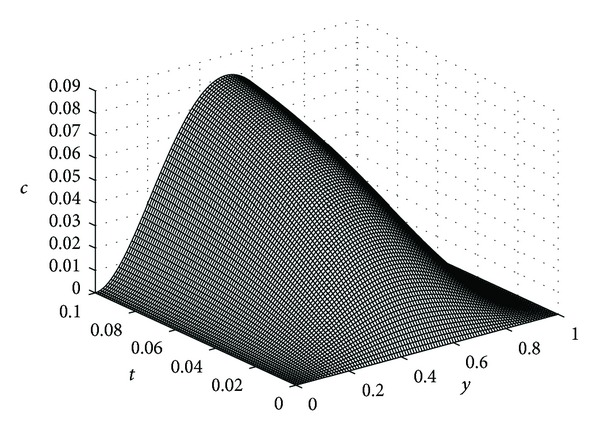
Proteolytic enzyme density *c*(*y*, *t*).

**Figure 5:**
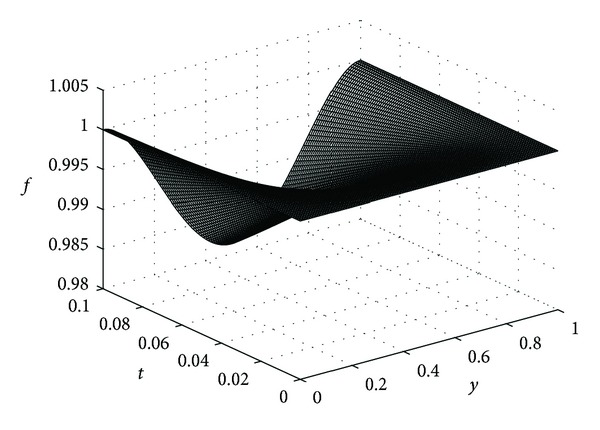
Fibronectin density *f*(*y*, *t*).

**Figure 6:**
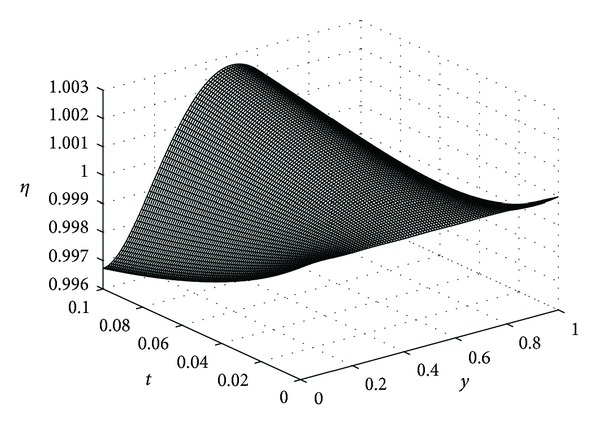
Endothelial cell density *η*(*y*, *t*).

Figure 1 and Figures 2–6 have been created using WinEdt/MiKTeX and MATLAB, respectively.

When running numerical computations, one might face the bimodality properties of endothelial cells as time increases. But, for what conditions this situation holds? This question has been addressed in [19], and there the authors have observed that if gradient ∂_*y*_ *v*(*y*, 0) is large and they start with a unimodal distribution in *v*(*y*, 0), a bimodal distribution in ln⁡*τ*(*c*, *f*) may develop. The bimodal structure of ln⁡*τ*(*c*, *f*) in turn leads to the bimodality of *η*, the EC distribution, and provides evidence that our model is in agreement with biology since the bimodal EC distribution correspond to the beginnings of the EC lining of the nascent capillary. As the reader remembers, in our numerical example we take *v*(*y*, 0) = *ϵθ*(*y*) = *ϵ* *Ay* ^2^(1 − *y*)^2^. Even though this function is of unimodal distribution, gradient ∂_*y*_ *v*(*y*, 0) is not sufficiently large to develop the bimodality of *η*. One could, of course, choose *v*(*y*, 0) = *ϵ* *Ay* ^*m*^(1 − *y*)^*m*^ for some *m* > 0 and try to find the desired constant *m* which creates the bimodality of *η*. According to our numerical experience, finding such constant *m* may not be easy.

We must also mention the importance of the function *θ*(*y*) appearing in the initial data. It is a function that initiates the dynamics in the model equations. If it is zero, every variable in the model stays dormant, and no capillary forms for angiogenesis to occur.

On the other hand, we have determined the stability criteria for the steady-state solution of the endothelial cell equation. Although we do not present the pictures in the paper, all of the four variables (*v*, *c*, *f*, *η*) come to steady state, about *t* = 25.

We are in the process of expanding the idea used in this paper to our 2D problem (in ECM, see Figure 1). The results obtained here look promising that we will have similar results for cells in ECM, where a capillary branches from a given capillary and lining itself with endothelial cells as it progresses.

## References

[1] Paweletz N, Knierim M (1989). Tumor-related angiogenesis. *Critical Reviews in Oncology/Hematology*.

[2] Nicosia RF, Bonanno E, Smith M (1993). Fibronectin promotes the elongation of microvessels during angiogenesis in vitro. *Journal of Cellular Physiology*.

[3] Yamada KM, Olden K (1978). Fibronectins—adhesive glycoproteins of cell surface and blood. *Nature*.

[4] Folkman J (1971). Tumor angiogenesis: therapeutic implications. *New England Journal of Medicine*.

[5] Folkman J (1976). The vascularization of tumors. *Scientific American*.

[6] Schleef RR, Birdwell CR (1982). The effect of proteases on endothelial cell migration in vitro. *Experimental Cell Research*.

[7] Othmer HG, Stevens A (1997). Aggregation, blowup, and collapse: the ABC’S of taxis in reinforced random walks. *SIAM Journal on Applied Mathematics*.

[8] Pamuk S (2003). Qualitative analysis of a mathematical model for capillary formation in tumor angiogenesis. *Mathematical Models and Methods in Applied Sciences*.

[9] Schor AM, Schor SL (1983). Tumour angiogenesis. *Journal of Pathology*.

[10] Bellomo N, Li NK, Maini PK (2008). On the foundations of cancer modelling: selected topics, speculations, and perspectives. *Mathematical Models and Methods in Applied Sciences*.

[11] Bellomo N, Bellouquid A, Nieto J, Soler J (2012). On the asymptotic theory from microscopic to macroscopic growing tissue models: an overview with perspectives. *Mathematical Models and Methods in Applied Sciences*.

[12] Bellouquid A, De Angelis E (2011). From kinetic models of multicellular growing systems to macroscopic biological tissue models. *Nonlinear Analysis: Real World Applications*.

[13] Zheng Z, Koh GY, Jackson T (2013). A continuous model of angiogenesis initiation, extention, and maturation of new blood vessels modulated by vascular endothelial
growth factor, angiopoietins, platelet-derived growth factor-b and Pericites. *Discrete and Continuous Dynamical Systems*.

[14] Pamuk S (2004). Steady-state analysis of a mathematical model for capillary network formation in the absence of tumor source. *Mathematical Biosciences*.

[15] Calvo J, MazÓn J, Soler J, Verbeni M (2011). Qualitative properties of the solutions of a nonlinear flux-limited equation arising in the transport of morphogens. *Mathematical Models and Methods in Applied Sciences*.

[16] Verbeni M, Sánchez O, Mollica E (2013). Morphogenetic action through flux-limited spreading. *Physics of Life Reviews*.

[17] Levine HA, Pamuk S, Sleeman BD, Nilsen-Hamilton M (2001). Mathematical modeling of capillary formation and development in tumor angiogenesis: penetration into the stroma. *Bulletin of Mathematical Biology*.

[18] Davis B (1990). Reinforced random walk. *Probability Theory and Related Fields*.

[19] Levine HA, Sleeman BD, Nilsen-Hamilton M (2001). Mathematical modeling of the onset of capillary formation initiating angiogenesis. *Journal of Mathematical Biology*.

[20] Levine HA, Sleeman BD, Nilsen-Hamilton M (2000). A mathematical model for the roles of pericytes and macrophages in the initiation of angiogenesis—I. The role of protease inhibitors in preventing angiogenesis. *Mathematical Biosciences*.

[21] Berg PW, McGregor JL (1966). *Elementary Partial Differential Equations*.

